# *Rosa roxburghii* fermented juice mitigates LPS-induced acute lung injury by modulation of intestinal flora and metabolites

**DOI:** 10.3389/fmicb.2024.1447735

**Published:** 2024-09-17

**Authors:** Zhiyu Chen, Shuo Zhang, Xiaodong Sun, Duo Meng, Chencen Lai, Min Zhang, Pengjiao Wang, Xuncai Huang, Xiuli Gao

**Affiliations:** ^1^State Key Laboratory of Functions and Applications of Medicinal Plants, School of Pharmaceutical Science, Guizhou Medical University, Guiyang, China; ^2^Center of Microbiology and Biochemical Pharmaceutical Engineering, Guizhou Medical University, Guiyang, China; ^3^Experimental Animal Center of Guizhou Medical University, Guiyang, China; ^4^Guizhou Provincial Engineering Research Center of Food Nutrition and Health, Guizhou Medical University, Guiyang, China

**Keywords:** *Rosa roxburghii* fermented juice, metabolomics, intestinal flora, *Rosa roxburghii*, *Rosa roxburghii* Tratt

## Abstract

Acute lung injury (ALI) is a severe pulmonary condition with high mortality and morbidity, lacking effective pharmacotherapeutic options. *Rosa roxburghii* Tratt, a unique fruit from southwestern China, is valued for its rich nutritional content and functional properties. Fermentation is known to enhance the nutritional value, flavor, and shelf life of foods. In this study, we investigated the effects of fermented *Rosa roxburghii* juice (RRFJ) on gut microbiota, metabolites, and the levels of short-chain fatty acids in the intestines, as well as its impact on lung tissue and intestine tissue injury, inflammation, and oxidative stress in murine models. The results showed that RRFJ modulated gut microbiota and metabolites, increased short-chain fatty acid levels, and consequently reduced lung tissue injury, inflammation, and oxidative stress in mice with ALI. These findings suggest that RRFJ has the potential to serve as a functional dietary adjunct in the management of acute lung injury, providing a scientific basis for its therapeutic role.

## 1 Introduction

Acute lung injury (ALI) is a severe respiratory disease characterized by diffuse noncardiogenic pulmonary edema resulting from alveolar injury (Lai et al., 2023). ALI arises from a range of diseases and diverse causal factors including sepsis, pneumonia, infection, trauma, ischemia–reperfusion, drug toxicity, among others (Shang et al., 2024). Glucocorticoids are commonly used in clinical research to treat ALI, but they entail numerous side effects including skin atrophy, decreased bone density, and gastrointestinal discomfort (Zhu et al., 2023; Sun et al., 2024). Consequently, due to the absence of specific drug treatments, ALI often progresses to its severe form, acute respiratory distress syndrome (ARDS). ARDS is a clinical syndrome characterized by a high mortality rate ranging from 35 to 46% (Bellani et al., 2016). According to a global literature survey, the mortality rate of COVID-19-related ARDS in 2019 was 45%, with an incidence rate of 90% among non-survivors of COVID-19 (Tzotzos et al., 2020). ALI can result from various pathogenic factors, including direct and indirect injuries induced by pronounced inflammation (De Freitas Caires et al., 2018). As most researchers consider the inflammatory response to be central to ALI, treatment continues to emphasize effectively managing its degree (Liu et al., 2021). Despite advancements, specific drugs remain elusive, prompting exploration into functional foods as adjunctive treatments for ALI.

*Rosa Roxburghii Tratt* (RRT) is a wild deciduous shrub belonging to the Rosa genus of Rosaceae family (Wang et al., 2023). It is recognized as a high-quality fruit with the third generation of homology of medicine and food in China. RRT is primarily distributed in southwest China, particularly in Guizhou Province, where it is locally known as “cili.” Numerous scientific studies have demonstrated that RRT contains a wide array of nutrients and active compounds, including polysaccharides, proteins, polyphenols, triterpenes, organic acids, and various essential amino acids, with particular richness in vitamin C and superoxide dismutase (Li-Tao Wang et al., 2021). Consequently, RRT finds wide application in the production of beverages and food products. In our preliminary research, our team discovered that polyphenols in RRT have a protective effect on mice with acute lung injury induced by LPS (Tang et al., 2023). Nearly half of Guizhou Province’s annual RRT production is processed into dried fruit, but the taste and nutritional value of the dried fruit are inferior to that of fresh RRT. Fermentation is an effective method to enhance the nutritional quality and flavor of food. After fermentation, the total phenol content of *Rosa roxburghii* Tratt juice increases, along with enhanced antioxidant and xanthine oxidase inhibition capabilities (Zhou et al., 2023). Additionally, it has the potential to boost immunity, reduce intestinal damage, and modulate the gut microbiota (Xu et al., 2024). Nevertheless, it remains uncertain whether *Rosa roxburghii* fermented juice (RRFJ) confers a preventive effect on ALI. Hence, this study will furnish a critical scientific foundation for the advancement of RRFJ as a functional food for ALI prevention.

This study investigates RRFJ for its therapeutic potential in lipopolysaccharide (LPS)-induced ALI in mice. Male Balb/c mice were divided into Control, LPS, Dexamethasone (DEX), and RRFJ groups. LPS was used to induce the ALI model. We employed a comprehensive approach integrating histopathology, cytokine assessment, metabolomics, gut microbiota analysis, and short-chain fatty acids (SCFAs) determination. We aim to uncover RRFJ’s mechanisms in ALI via lung-gut axis, including its effects on inflammation, oxidative stress, metabolites, gut microbiota, and SCFAs production.

## 2 Materials and method

### 2.1 Materials and reagents

*Rosa roxburghii* Fermented Juice (Production date: 2022-12-31, batch number: Q/SWG0001S) was supplied by Guizhou Shanwangguo Health Industry Co. Ltd. (Guizhou, China). LPS (*Escherichia coli* serotype 055) was acquired from Sigma-Aldrich. Dexamethasone sodium phosphate injection (DEX, 5 mg/mL, batch number 22009281) was obtained from Suicheng Pharmaceutical Co. Ltd. (Zhengzhou, China). ELISA kits for TNF-α, IL-10, IL-6, and IL-1β were sourced from Shenzhen NeoBioscience Co. Ltd. (China). Assay kits for MDA, SOD, GSH, and MPO were purchased from Nanjing Jiancheng Bioengineering Institute. UPLC-grade reagents (formic acid, acetonitrile, and methanol) were obtained from Merck, while all other chemicals and reagents were of analytical grade. The primary antibodies used were: TNF-α (17590-1-AP), IL-10 (82191-3-RR), and IL-6 (21865-1-AP) from Proteintech (Wuhan, China), and IL-1β (#63124S) from Cell Signaling Technology (Boston, United States).

### 2.2 Animals and experimental design

**Figure 1:**
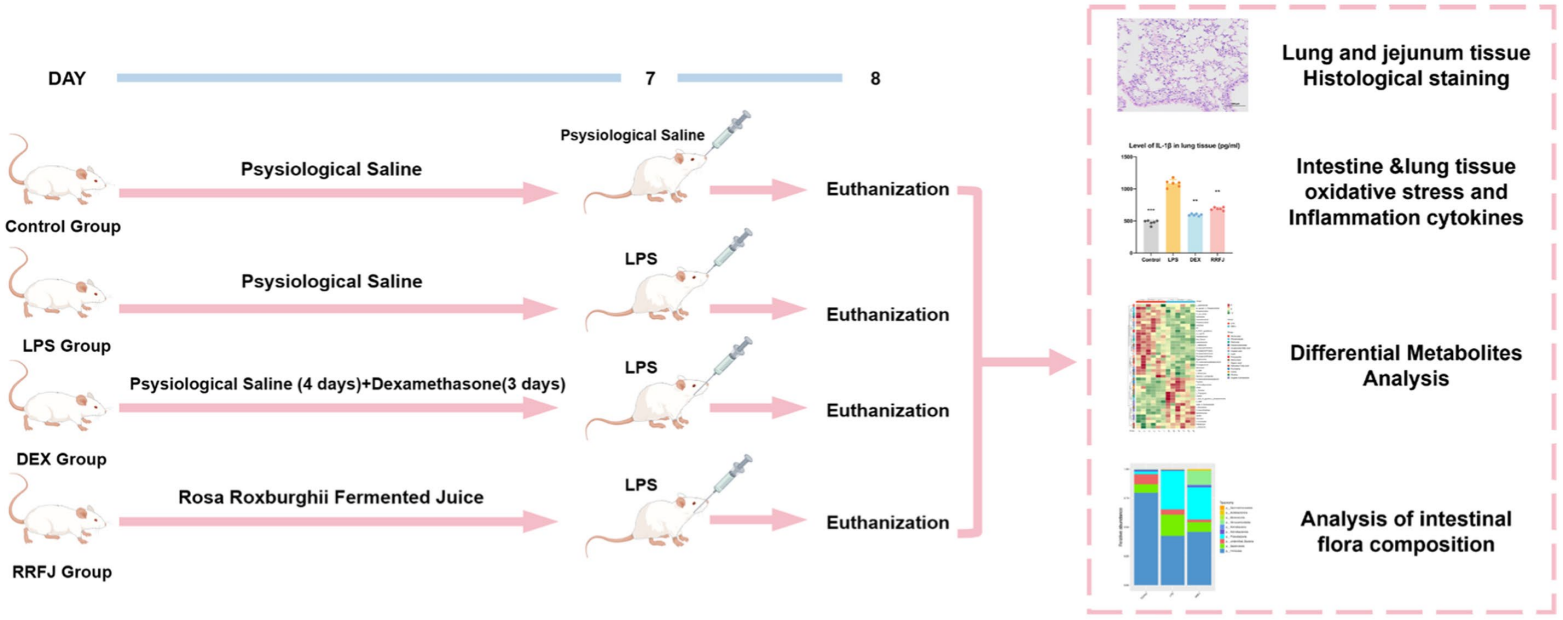
Schematic diagram of experimental design.

A total of 24 male BALB/C mice, weighing 18-22 g, were sourced from the Animal Centre of Guizhou Medical University (SPF grade, License no. SCXK (xiang) 2022-0011) (Changsha Tianqin Biotechnology Co. LTD, Hunan, China). The mice were housed under standard laboratory conditions, maintaining a 12-h light/dark cycle, temperature of 25 ± 1°C, and humidity of 60 ± 5%, with unrestricted access to water and food. The animal study adhered strictly to the “Guidelines for the Care and Use of Experimental Animals” and received approval from the Ethics Committee of Guizhou Medical University [license no. SYXK (qian) 2018-0001]. After a week of acclimation, the mice were randomly assigned to four groups: control, LPS, Dexamethasone (DEX), and RRFJ (LPS + RRFJ 20 mL/kg). The control and LPS groups received saline gavage for 7 days. The DEX group was administered saline gavage for 4 days, followed by intraperitoneal dexamethasone (2 mg/kg/day) for 3 days. The RRFJ group received RRFJ by gavage for 7 days. The ALI model was induced using LPS. Mice were anesthetized with an intraperitoneal injection of 0.3% pentobarbital sodium, followed by oropharyngeal LPS administration (2 mg/kg) with 30 s of nasopharyngeal occlusion. The control group received saline instead of LPS. Mice were euthanized 24 h post-LPS administration (Figure 1).

### 2.3 Histopathological analysis

Mouse lung and jejunum tissues were fixed in 4% paraformaldehyde, then dehydrated, embedded in paraffin, and sectioned into 4 μm slices using a Leica rotary microtome. The sections were stained with hematoxylin–eosin (H&E) and examined under a light microscope.

### 2.4 Cytokine concentration measurement

Levels of tumor necrosis factor-α (TNF-α), interleukin-6 (IL-6), interleukin-1β (IL-1β), and interleukin-10 (IL-10) in lung and intestinal tissues were measured using ELISA kits, following the manufacturer’s instructions.

### 2.5 MDA, SOD, GSH, and MPO analysis

Malondialdehyde (MDA) levels, superoxide dismutase (SOD) activity, glutathione (GSH) levels, and myeloperoxidase (MPO) levels in lung and intestinal tissues were quantified using assay kits from Nanjing Jiancheng Bioengineering Institute.

### 2.6 Western blot analysis

Lung and intestinal tissues were lysed in a buffer containing 1% PMSF on ice for 2 h. Lysates were centrifuged at 12,000 g for 10 min at 4°C, and supernatants were collected. Proteins were separated by 10% SDS-PAGE and transferred to polyvinylidene fluoride (PVDF) membranes. The membranes were blocked with 5% skim milk for 1 h, then incubated with primary antibodies overnight at 4°C. Following incubation with secondary antibodies for 1 h, protein bands were visualized using the Tanon 5200 ECL detection system (Tanon, China) and analyzed semi-quantitatively with ImageJ software.

### 2.7 Metabolomics analysis

A 100 mg fecal sample was extracted using 0.5 mL of precooled aqueous methanol/acetonitrile solution (2:2:1, v/v/v), followed by homogenization and low-temperature vortex-sonication for 15 min. Protein precipitation was achieved by storing the mixtures overnight at −20°C, then centrifuging at 15,000 rpm for 15 min, and collecting the supernatants. The filtered supernatants (0.22 μm membrane) were prepared for metabolomics analysis. Metabolomics was conducted using UHPLC-ESI-Q-Exactive Plus Orbitrap-MS, equipped with a ZORBAX Eclipse Plus C18 column (2.1 × 100 mm, 1.8 μm; Agilent Technologies, United States). The optimized gradient used was as follows: 0–2.5 min, 2–2% B; 2.5–5 min, 2–40% B; 5–12 min, 40–100% B; 12–16 min, 100–100% B; 16–16.1 min, 100–2% B; 16.1–19 min, 2–2% B, with a flow rate of 0.3 mL/min. Ion spray voltage was set to 3.5/2.8 kV (+/−), with a scan range of 100–1,500 m/z. Auxiliary gas heater and capillary temperatures were 350°C and 320°C, respectively. Dynamic exclusion was set at 3 s, with an S-lens RF level of 50. MS data preprocessing, including peak identification, matching, and retention time alignment, was performed using Compound Discoverer 3.2 software. SIMCA-P 14.1 (Umetrics, AB, Sweden) was utilized for OPLS-DA and PCA. Endogenous metabolites were identified by comparing primary and secondary mass spectra using KEGG^1^ and HMDB^2^ databases, supplemented by in-house MS2 spectral library verification. MetaboAnalyst 5.0 facilitated the analysis of metabolic pathways implicated in ALI following RRTP preventive treatment, with significant pathways defined by an impact value > 0.1.

### 2.8 Gut microbiota analysis

CTAB or SDS method is used to extract the genome DNA of the sample, and then the purity and concentration of DNA are detected by agarose gel electrophoresis. An appropriate amount of DNA is taken in the centrifuge tube and the sample is diluted to 1 ng/μl with sterile water. Using diluted genome DNA as a template, according to the selection of sequencing areas, special primers with Barcode, Phusion^®^ High-Fidelity PCR Master Mix with GC Buffer of New England Biolabs, and high-efficiency fidelity enzymes are used for PCR to ensure amplification efficiency and accuracy. PCR amplification of bacterial 16 s rRNA gene V3-V4 region was performed using the forward primers 515F (5′-GTGCCAGCMGCCGCGGTAA-3′) and reverse primers 806R (5′-GGACTACHVGGGTWTCTAAT-3′). The PCR product used 2% concentration of agarose gel for electrophoresis detection, then purified the qualified PCR products, and then used enzyme standard quantification to mix an equal amount according to the concentration of PCR products. After full mixing, 2% agarose gel electrophoresis was used to detect PCR products. Finally, the rubber recovery kit provided by Qiagen was used to recycle the target strip. The TruSeq^®^ DNA PCR-Free Sample Preparation Kit was used to build the library. The constructed library had been quantified by Qubit and Q-PCR. After the library was qualified, NovaSeq6000 was used for on-machine sequencing. After removing the Barcode and primer sequences, the 250 bp paired-end reads obtained from sequencing were merged using FLASH to obtain raw data (Raw Tags). The Raw Tags were then subjected to stringent filtering according to the quality control process of the Qiime (V1.9.1) platform to obtain clean data (Clean Tags). All effective tags were subjected to clustering analysis using Uparse (v7.0.1001), with sequences having a default consistency greater than 97% clustered into operational taxonomic units (OTUs), representing microbial species. Subsequently, species annotation analysis of OTUs was performed using the SSUrRNA database (threshold of 0.8-1). After data normalization, α-diversity analysis was conducted using Qiime software (Version 1.9.1). Principal coordinates analysis (PCoA) based on Weighted UniFrac distance was performed to represent β-diversity analysis differences.

### 2.9 Determinations of short-chain fatty acids in fecal contents

Two hundred milligrams of caecal contents were mixed with 1 mL of ultrapure water and centrifuged at 1,000 rpm for 10 min using an ALLEGRA-64R centrifuge (Beckman Coulter, United States). The supernatant was filtered, then 7 μL of 50% sulfuric acid and 1 mL of ether were added. The ether layer was collected in a sample vial for GC analysis. Short-chain fatty acids (SCFAs) were analyzed using an Agilent 7980A gas chromatograph (Agilent Technologies, Santa Clara, United States) with a flame ionization detector (FID) and an InertCap WAX GC column (30 m × 0.53 mm i.d., 1 μm film thickness, Agilent). High-purity nitrogen served as the carrier gas. The injection was performed using a splitless mode with a 0.8 μL sample volume. The temperature program was as follows: 80°C (1 min), increased to 115°C at 15°C/min (3 min hold), increased to 130°C at 3°C/min (no hold), then increased to 230°C at 15°C/min (3 min hold). The FID detector was set to 250°C. SCFA concentrations were quantified using an external standard method.

### 2.10 Statistical analysis

This experimental data is analyzed using SPSS 16.0 and GraphPad Prism 8.0 software and expressed as an average ± standard deviation. When *p* < 0.05, the statistical significance is significant. In addition, the statistical significance is *p* < 0.01, and the statistical significance is very significant.

## 3 Results

### 3.1 Effects of RRFJ on histological change in lung tissue on ALI mice

**Figure 2:**
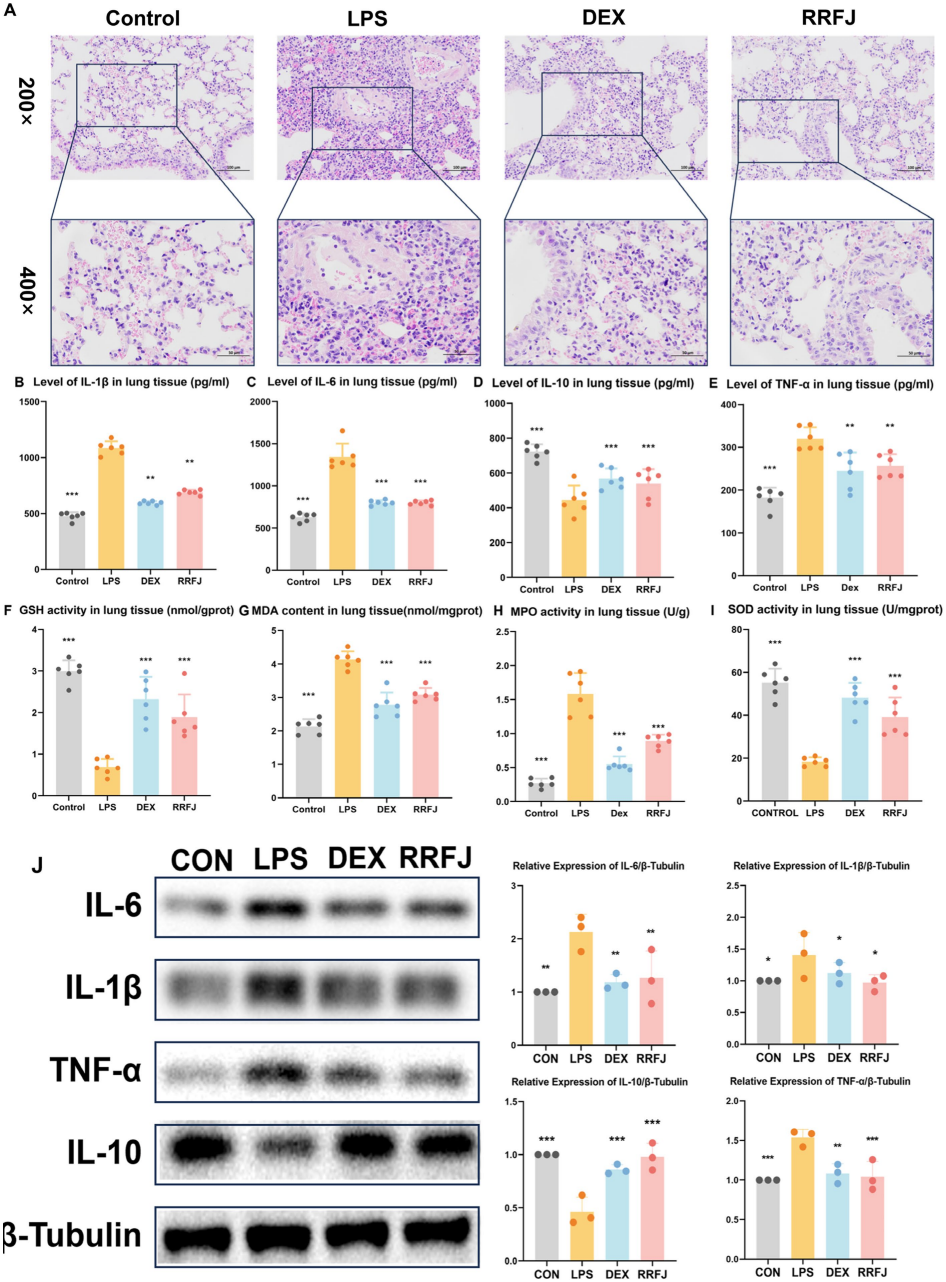
Effects of RRFJ on pathological changes to lung tissue on cytokine concentration and oxidative stress in lung tissue in LPS-induced ALI model mice. **(A)** Pathological changes in lung tissues (Magnification = 200× and 400×). **(B–E)** Effects of RRFJ on cytokine concentration of lung tissue in ALI mice. **(F–I)** Effects of RRFJ on the level of oxidative stress of intestine tissue in ALI mice. **(J)** The protein expression levels of cytokine concentration in lung tissue were detected by Western blotting, *n* = 3.Compared with the LPS group, *p < 0.05, **p < 0.01, ***p < 0.001.

To scrutinize the impact of RRFJ on the morphological and histological alterations in lung tissue 24 h post-LPS injection, the morphological and histological changes in murine pulmonary tissue were evaluated utilizing Hematoxylin–Eosin (H&E) staining. As illustrated in Figure 2A, oropharyngeal inhalation of normal saline did not induce notable morphological damage to the lungs of healthy mice. LPS-induced ALI mice manifested pronounced inflammatory cell infiltration, marked thickening of the alveolar wall, and pulmonary hyperemia. However, following administration of RRFJ and DEX, the pulmonary pathology severity in ALI mice exhibited marked amelioration. These findings elucidate the protective efficacy of RRFJ against LPS-induced ALI.

### 3.2 Effects of RRFJ on histological change in jejunum tissue on ALI mice

**Figure 3:**
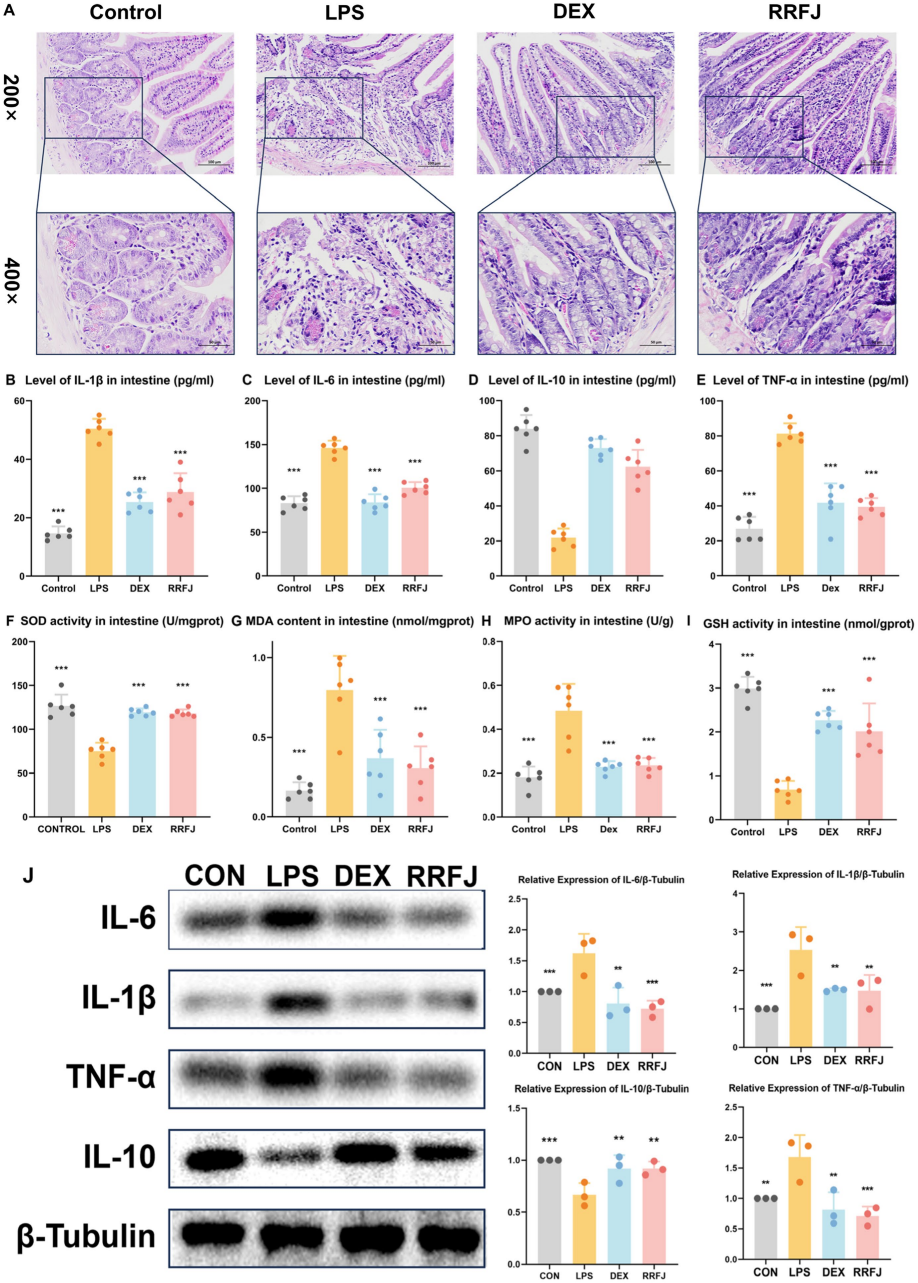
Effects of RRFJ on pathological changes jejunum tissue and on cytokine concentration and oxidative stress in intestine tissue in LPS-induced ALI model mice. **(A)** Pathological changes in jejunum tissues (Magnification = 200× and 400×). **(B–E)** Effects of RRFJ on cytokine concentration of intestine tissue in ALI mice. **(F–I)** Effects of RRFJ on the level of oxidative stress of intestine tissue in ALI mice. **(J)** The protein expression levels of cytokine concentration in intestine tissue were detected by Western blotting, *n* = 3. Compared with the LPS group, *p < 0.05, **p < 0.01, ***p < 0.001.

Using H&E staining, we performed a comprehensive morphological and histological examination of the jejunum tissue in ALI mice (Figure 3A). In the H&E-stained sections of the LPS group, we observed pathological changes, including focal erosion in some jejunum tissues, slight edema in the submucosal layer, reduced numbers of lymphocytes and granulocytes, and an increased presence of Paneth cells. However, treatment with DEX or RRFJ significantly ameliorated these severe histopathological changes. These findings highlight the alleviating effects of RRFJ on the pathological damage to jejunum tissue in ALI mice.

### 3.3 Effects of RRFJ on cytokine concentration of lung tissue and intestinal tissue

To investigate the impact of RRFJ on the inflammatory response, levels of pro-inflammatory cytokines (IL-6, IL-1β, TNF-α) and the anti-inflammatory cytokine IL-10 in intestinal tissues and lung tissues were quantified. As shown in Figures 2B–E, 3B–E, LPS significantly enhanced the secretion of IL-6, IL-1β, and TNF-α, while reducing the synthesis of IL-10 in both lung and intestinal tissues. However, following treatment with DEX or RRFJ, these changes were reversed. Western blot analysis demonstrated that the protein expression levels of IL-6, IL-1β, and TNF-α were significantly upregulated, while IL-10 was notably downregulated in the lung tissue and intestine tissue of LPS-induced ALI mice. However, this effect was reversed following treatment with DEX and RRFJ (Figures 2J, 3J). These results indicated that RRFJ attenuated inflammation in LPS-induced ALI mice.

### 3.4 RRFJ inhibits oxidative stress in LPS-induced ALI mice

Additionally, oxidative stress plays a critical role in ALI, ultimately leading to its progression to ARDS. Thus, to elucidate the role of RRFJ in the response to oxidative stress, we assessed the activities of SOD, MPO, and GSH, as well as the concentration of MDA in both lung and intestinal tissues of ALI-afflicted mice. As illustrated in Figures 2F–I, 3F–I, LPS elevated MDA levels, increased MPO activity, and suppressed GSH and SOD activities in both lung and intestinal tissues. However, following treatment with RRFJ, the MDA content and MPO activities were reduced, and the GSH and SOD activities were increased, suggesting that RRFJ mitigated the oxidative stress response in the intestinal tissue of LPS-induced ALI mice.

### 3.5 Fecal metabolism analysis

**Figure 4:**
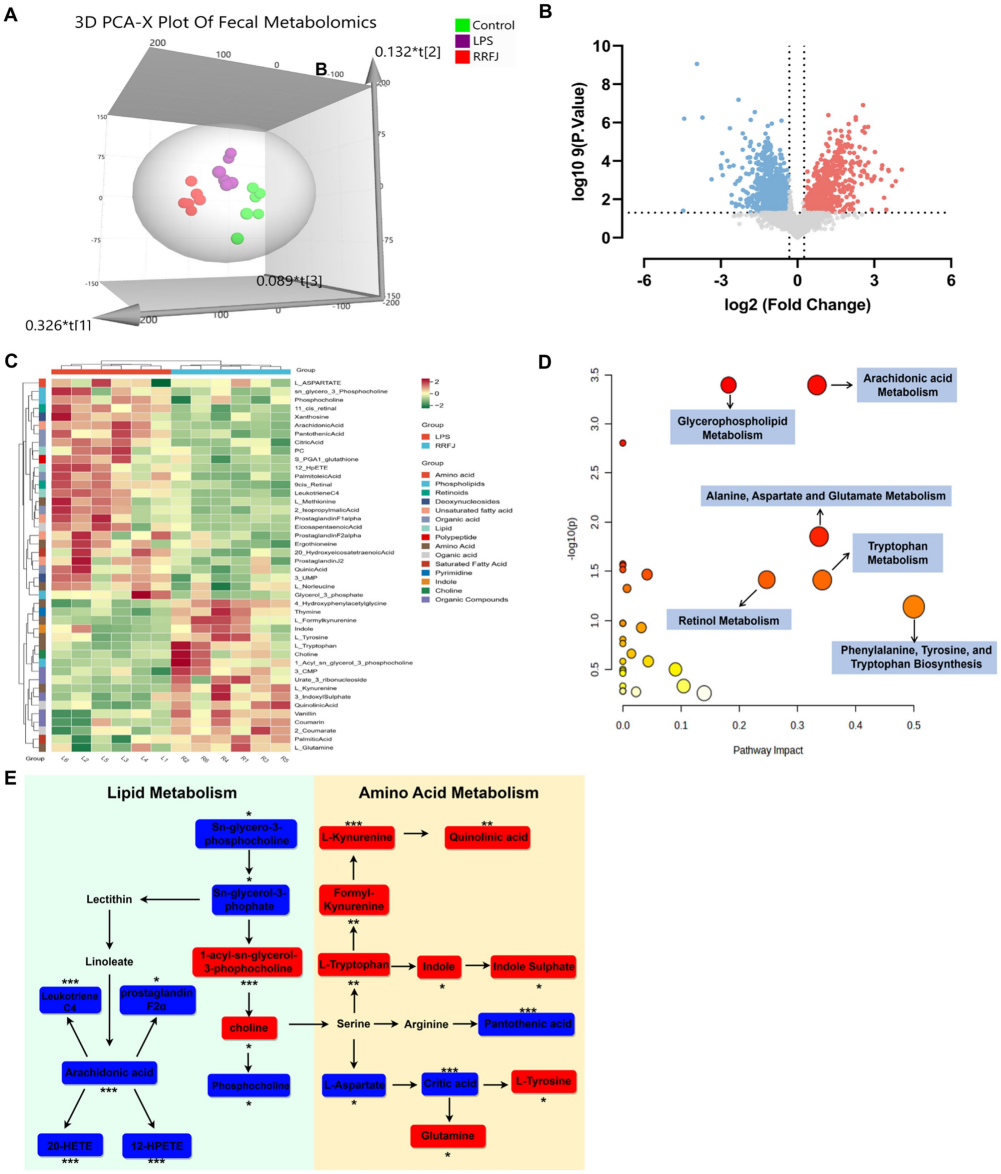
RRFJ modulated the fecal metabolome. **(A)** 3D-PCA diagram of cecal contents. **(B)** Volcano gram analysis of differential metabolites in fecal. **(C)** Heatmap of differentially expressed metabolites in cecal contents. **(D)** Enrichment analysis of differential expressed metabolites in cecal contents. **(E)** Representative metabolism showed in metabolomics analysis, blue represents pathways downregulated by RRFJ whereas red represents pathways upregulated by RRFJ. Compared with the LPS group, *p < 0.05, **p < 0.01, ***p < 0.001.

To further understand the protective mechanism of RRFJ against ALI, we employed UHPLC–MS/MS for fecal metabolomic analysis. Principal component analysis (PCA) revealed distinct separation among the control group, LPS group, and RRFJ group (Figure 4A). Volcano plot analysis was used to identify differential metabolites between the RRFJ group and the LPS group (Figure 4B). By applying screening criteria of VIP > 1, *p* < 0.05, and a fold change > 1.2 or < 0.8, we identified 79 metabolites. *p*-value of < 0.05 indicates statistical significance, suggesting that the differences between groups are unlikely to be due to random chance. Fold change greater than 1.2 or less than 0.8 indicates significant biological changes in metabolite levels. VIP value greater than 1 indicates that the metabolite makes an important contribution to the differentiation between groups in the OPLS-DA model. The heatmap showed discernible differences between the RRFJ group and the LPS group (Figure 4C). To elucidate the potential metabolic pathways affected by RRFJ in ALI, we conducted pathway analysis of the differential metabolites using the MetaboAnalyst 5.0 platform. Metabolic pathways with the greatest impact (Pathway Impact > 0.1) included phenylalanine, tyrosine, and tryptophan biosynthesis; tryptophan metabolism; alanine, aspartate, and glutamate metabolism; arachidonic acid metabolism; retinol metabolism; and glycerophospholipid metabolism (Figure 4D). The representative metabolic pathway is illustrated in Figure 4E.

### 3.6 Gut microbiota analysis

**Figure 5:**
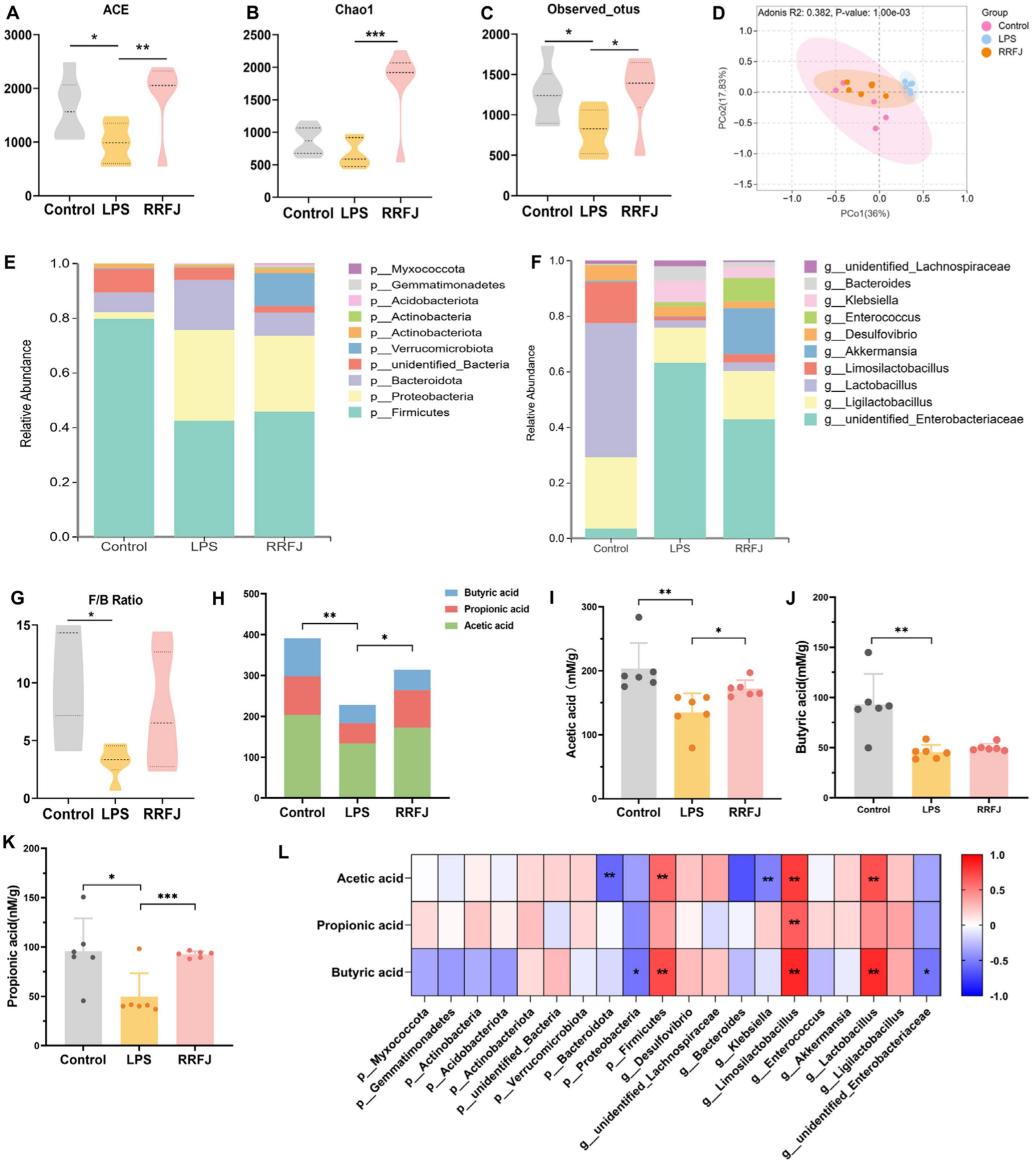
RRFJ modulated the gut microbiota and SCFAs. **(A)** The alpha-diversity of ACE index. **(B)** The alpha-diversity of Chao1 index. **(C)** The alpha-diversity of observed OTUs index. **(D)** Weighted UniFrac PCoA analysis of gut microbiota based on the ASVs data on Control, LPS and RRFJ group. **(E)** The average percent of community abundance on phylum level. **(F)** The average percent of community abundance on genus level. **(G)** Firmicutes/Bacteroidota ratio. **(H)** The level of total SCFAs. **(I)** The level of acetic acid. **(J)** The level of butyric acid. **(K)** The level of propionic acid. **(L)** Heat map of correlation between intestinal flora and SCFAs. Compared with the LPS group, *p < 0.05, **p < 0.01, ***p < 0.001.

To investigate the impact of RRFJ on the intestinal microbiome, we performed additional assessments to determine alterations in intestinal flora within each experimental group. Initially, we assessed the ecological diversity of microbial communities using alpha-diversity measures. Alpha-diversity metrics (Chao1 index, Shannon index, and observed OTUs index) revealed significant disparities in bacterial richness and diversity between the LPS and RRFJ groups (Figures 5A–C). Subsequently, we performed PCA analysis to assess beta-diversity between groups. As shown in Figure 5D, distinct separation was observed among the Control, RRFJ, and LPS groups in the PCA plot. At the phylum level (Figure 5E), compared to the Control group, the diversity of intestinal flora in the LPS group significantly decreased, with a reduction in Firmicutes abundance and the Firmicutes/Bacteroidota ratio, and an increase in the abundance of Proteobacteria and Bacteroidota. Following RRFJ treatment, an increase in intestinal flora diversity was observed, accompanied by an elevation in Verrucomicrobiota abundance and a decrease in Proteobacteria and Bacteroidota abundances. At the genus level (Figure 5F), compared to the Control group, the abundances of unidentified_Enterobacteriaceae, Bacteroides, and Klebsiella increased, while the abundances of Ligilactobacillus, Limosilactobacillus, and Akkermansia decreased. However, following RRFJ treatment, the opposite trends were observed. Overall, the intestinal flora in LPS-induced ALI exhibited dysbiosis, but RRFJ treatment ameliorated this condition, particularly with regard to the Firmicutes/Bacteroidota ratio (Figure 5G), Akkermansia, Ligilactobacillus, and Lactobacillus.

### 3.7 Analysis of short-chain fatty acids in fecal contents

As shown in Figure 5H, LPS significantly reduced the total amount of SCFAs compared to the control group (*p* < 0.01). In contrast, the SCFAs levels in the RRFJ group significantly increased compared to the LPS group (*p* < 0.05). RRFJ increased the content of acetic acid (from 134.8737 ± 30.0038 to 172.0798 ± 13.5813, *p* < 0.01), propionic acid (from 49.6464 ± 23.7981 to 92.5815 ± 3.21543, *p* < 0.001), and butyric acid (from 45.5613 ± 7.1983 to 50.0652 ± 3.8857) (Figures 5I–K). In addition, we evaluated the correlation between intestinal flora and SCFAs (Figure 5L). The results showed that SCFAs were positively correlated with Firmicutes, Limosilactobacillus, and Lactobacillus (*p* < 0.05), and negatively correlated with Bacteroidota, Proteobacteria, Klebsiella, and unidentified_Enterobacteriaceae (*p* < 0.05).

### 3.8 Integrated analysis of intestinal flora and metabolites

**Figure 6:**
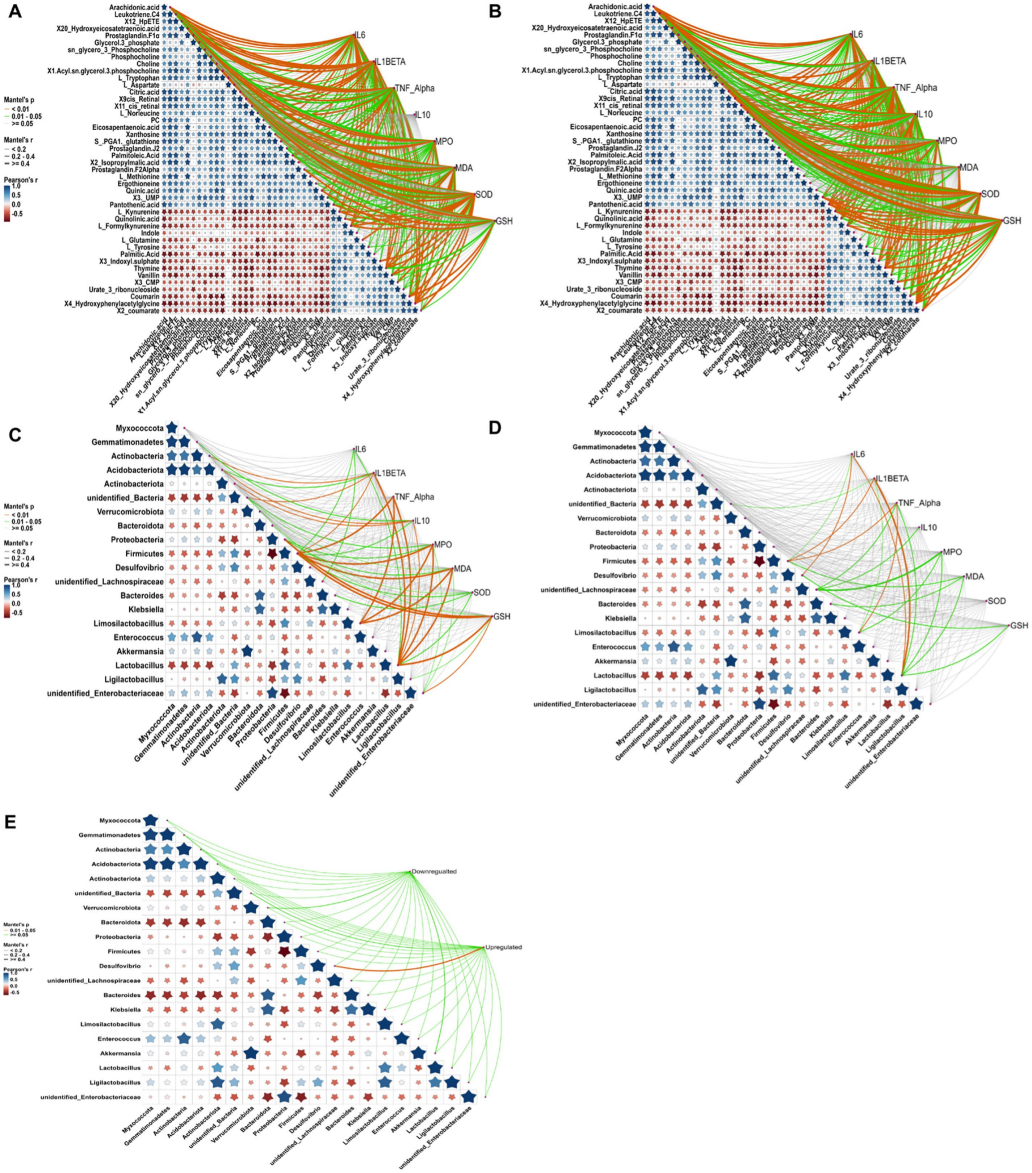
Correlation analysis between cytokine levels, oxidative stress, metabolites, and gut microbiota, conducted using the Mantel test in RStudio. Orange curves indicate significant correlations, green curves denote statistical differences, and gray curves represent non-significant correlations. The thickness of the curve correlates with the magnitude of the correlation coefficient; thicker curves signify a higher correlation coefficient. **(A)** Correlation analysis between metabolites, cytokines, and oxidative stress in lung tissue. **(B)** Correlation analysis between metabolites, cytokines, and oxidative stress in intestinal tissue. **(C)** Correlation analysis between gut microbiota, cytokines, and oxidative stress in lung tissue. **(D)** Correlation analysis between gut microbiota, cytokines, and oxidative stress in intestinal tissue. **(E)** Correlation analysis between gut microbiota and metabolites. Compared to the LPS group, *p < 0.05, **p < 0.01, ***p < 0.001.

To further elucidate the intricate mechanisms underlying RRFJ’s efficacy against ALI, we conducted a comprehensive analysis of the correlations between cytokine concentrations, oxidative stress levels, metabolites, and intestinal flora. Analysis of the associations between metabolites, cytokines, and oxidative stress levels in lung tissue revealed significant correlations with various amino acids and lipid metabolites. TNF-α, IL-10, and IL-6 levels in lung tissues demonstrated significant correlations with formyl-kynurenine, citric acid, leukotriene C4, and others (*p* < 0.01), and statistical relevance with L-tryptophan, L-tyrosine, and 12-HpETE (*p* < 0.05) (Figure 6A). Levels of SOD, GSH, MDA, and MPO in lung tissues showed statistical associations with 11cis-retinal and 9cis-retinal (*p* < 0.01). Specifically, MPO, SOD, and GSH exhibited significant correlations with arachidonic acid (*p* < 0.01), while SOD and GSH also displayed significant associations with L-kynurenine and formyl-kynurenine (*p* < 0.01). Mantel test results assessing the correlations between cytokines, oxidative stress levels, metabolites, and intestinal tissues revealed significant correlations between TNF-α, IL-10, IL-6, and IL-1β levels in intestinal tissues and L-kynurenine, arachidonic acid, 9cis-retinal, and 11cis-retinal (*p* < 0.01) (Figure 6A). Mantel test results assessing the correlations between cytokines, oxidative stress levels in lung and intestinal tissues, and metabolites indicated significant correlations between TNF-α, IL-10, IL-6, IL-1β levels in intestinal tissues and L-kynurenine, arachidonic acid, 9cis-retinal, and 11cis-retinal (*p* < 0.01). SOD and GSH levels in intestinal tissues were statistically related to L-kynurenine, arachidonic acid, and formyl-kynurenine (*p* < 0.05) (Figure 6B). The concentrations of cytokines and levels of oxidative stress in lung tissues were also closely related to intestinal flora. The results showed that cytokine concentrations and oxidative stress levels in lung tissues were statistically related to Firmicutes and Lactobacillus (*p* < 0.05) (Figure 6C). Mantel test results of cytokines and oxidative stress levels in intestinal tissues and intestinal flora showed that TNF-α, IL-6, and IL-1β were statistically correlated with Firmicutes and Lactobacillus, and MPO, MDA, and GSH were statistically related to Lactobacillus (*p* < 0.05) (Figure 6D). The analysis of the correlation between intestinal flora and metabolites revealed that the changes in upregulated and downregulated metabolites were closely related to intestinal flora (Figure 6E).

## 4 Discussion

This study thoroughly investigated the effects of RRFJ on gut microbiota and metabolites associated with inflammation in a mouse model of LPS-induced ALI. Although previous studies have demonstrated the potential of *Rosa roxburghii* Tratt polyphenols in alleviating ALI through various mechanisms, the specific effects of RRFJ on gut microbiota and metabolites in LPS-induced ALI are still unclear.

This study established an acute lung injury (ALI) model through LPS induction, demonstrating the efficacy of RRFJ in alleviating ALI. This is primarily evidenced by RRFJ’s attenuation of LPS-induced histological and pathological damage in both lung and intestinal tissues, as well as its ability to ameliorate TNF-α, IL-6, IL-1β, and IL-10 levels *in vivo*, thereby improving oxidative stress markers (MPO, SOD, GSH, MDA). Additionally, non-targeted metabolomics and microbial genomics analyses were utilized to systematically evaluate the specific effects of RRFJ on the composition and metabolic pathways of intestinal microbiota.

Through pharmacological experimentation, we have elucidated the multifaceted synergistic effects of RRFJ *in vivo*, including the reduction of lung congestion and alveolar wall thickening, the mitigation of focal erosion in jejunal tissue, a decrease in cytokine levels, and a reduction in oxidative stress within the organism. LPS, an extracellular membrane component of Gram-negative bacteria, has the potential to disrupt barrier integrity upon contact with pulmonary and intestinal tissues, thereby triggering the infiltration of numerous macrophages and inflammatory cells (Zhou et al., 2023; Tang et al., 2021). Oxidative stress can lead to excessive production of MPO and MDA within the organism, while simultaneously reducing the expression of antioxidant enzymes such as SOD and GSH, which are crucial for mitigating oxidative damage (Lv et al., 2017). Consequently, RRFJ supplementation significantly alleviates inflammation and oxidative stress in ALI mice, preserves intestinal barrier integrity, and maintains normal metabolic functions.

LPS-induced dysregulation of amino acids and lipids was observed in ALI mice. RRFJ modulates lipid metabolism in ALI mice, including the pathways of glycerophospholipid and arachidonic acid metabolism. Choline enhances immune tolerance in dendritic cells, promotes Treg cell differentiation, plays a critical role in immune function, and maintains cell membrane integrity (Li et al., 2022). As a primary component of cell membranes, arachidonic acid undergoes multiple metabolic transformations that elicit various inflammatory responses (Wang et al., 2019). Leukotriene C4 contributes to the exacerbation of pulmonary fibrosis in mice. Additionally, prostaglandin F2α, 20-HETE, and 12-HPETE, which are downstream metabolites of arachidonic acid, further exacerbate the inflammatory response during the ALI process (Hamers et al., 2022; Basu, 2007). In amino acid metabolism, the tryptophan metabolic pathway is crucial for maintaining intestinal homeostasis. The tryptophan metabolic pathway is associated with intestinal homeostasis within the broader context of amino acid metabolism. Tryptophan exerts a significant regulatory effect on LPS-induced ALI in mice, and its supplementation markedly reduces peroxide levels and neutrophil infiltration (Liu et al., 2020). Tryptophan metabolites, including kynurenine and indole, activate AhR receptors in the intestine, participate in immune regulation, and thereby protect the intestinal barrier in mice (Proietti et al., 2020; Sun et al., 2019). Glutamine, the most prevalent amino acid in the human body, stimulates intestinal cell proliferation, inhibits pro-inflammatory pathways, and protects cells from apoptosis and intestinal stress (Kim and Kim, 2017). These results underscore the protective effect of RRFJ against LPS-induced ALI.

RRFJ modulates lipid metabolism in ALI mice, including glycerophospholipid and arachidonic acid pathways. Choline enhances immune tolerance in dendritic cells, promotes Treg cell differentiation, plays a crucial role in immune function, and maintains cell membrane integrity (Li et al., 2022). Arachidonic acid, a primary component of cell membranes, undergoes various metabolic transformations that elicit diverse inflammatory responses (Wang et al., 2019). Leukotriene C4 contributes to the exacerbation of pulmonary fibrosis in mice. Additionally, prostaglandin F2α, 20-HETE, and 12-HPETE, which are downstream metabolites of arachidonic acid, further exacerbate the inflammatory response during ALI (Hamers et al., 2022; Basu, 2007). In amino acid metabolism, the tryptophan metabolic pathway is crucial for maintaining intestinal homeostasis. Tryptophan exerts a significant regulatory effect on LPS-induced ALI in mice, and its supplementation markedly reduces peroxide levels and neutrophil infiltration in ALI (Liu et al., 2020). Tryptophan metabolites, including kynurenine and indole, activate AhR receptors in the intestine, participate in immune regulation, and thereby protect the intestinal barrier in mice (Proietti et al., 2020; Sun et al., 2019). Glutamine, the most prevalent amino acid in the human body, stimulates intestinal cell proliferation, inhibits pro-inflammatory pathways, and protects cells from apoptosis and intestinal stress (Kim and Kim, 2017). These results underscore the protective effect of RRFJ against LPS-induced ALI.

The research findings indicate that RRFJ administration significantly increases the abundance of intestinal microbiota in ALI mice. At the phylum level, Firmicutes and Bacteroidetes are identified as the predominant bacterial taxa. RRFJ significantly increases the Firmicutes to Bacteroidetes (F/B) ratio in ALI mice. Multiple lines of evidence indicate that a significant reduction in the F/B ratio is a predictor of intestinal inflammation onset (Stojanov et al., 2020). RRFJ reduces the abundance of Proteobacteria in the intestines of ALI mice, suggesting its potential to alleviate the accumulation of pathogenic bacteria. Proteobacteria include various pathogenic bacteria such as *Escherichia coli* and *Helicobacter pylori*. Their accumulation in the intestines can lead to enteritis, intestinal stress syndrome, and metabolic disorders (Shin et al., 2015). RRFJ also improves the bacterial composition at the genus level in the intestines. RRFJ effectively alters the abundance of Enterobacteriaceae, Prevotella, Klebsiella, Lactobacillus, Limosilactobacillus, and Akkermansia in the intestines. Akkermansia provides protective benefits to gastrointestinal health and enhances the host’s immune system, metabolism, and intestinal barrier (Effendi et al., 2022; Niu et al., 2024). Lactobacillus effectively improves the intestinal microbiota and strengthens the intestinal barrier (Yadav et al., 2017; Miao et al., 2024). Spearman correlation analysis of phylum and genus levels with short-chain fatty acids (SCFAs) revealed significant positive correlations between SCFAs and Firmicutes, Lactobacillus, and Limosilactobacillus, as well as negative correlations with Bacteroidetes, Proteobacteria, and Klebsiella. SCFAs are strongly associated with the intestinal barrier. Acetic acid, propionic acid, and butyric acid account for 95% of the short-chain fatty acids in the intestine and serve as the primary energy source for intestinal epithelial cells. This metabolic function not only activates the intestinal barrier but also modulates the pulmonary immune response (Hou et al., 2022; Sivaprakasam et al., 2016; Sun et al., 2017). Increased levels of SCFAs promote the differentiation of Treg cells within the intestinal environment, thereby reducing the expression of IL-1β, TNF-α, and IL-6, and simultaneously enhancing IL-10 production (Atarashi et al., 2011; Atarashi et al., 2013). Moreover, SCFAs inhibit the phosphorylation of signal transduction factors and transcriptional activation molecules (STAT3), thereby reducing systemic oxidative stress (Tong et al., 2016). Therefore, these findings confirm that RRFJ improves intestinal microbial homeostasis in ALI mice by modulating gut microbiota composition and short-chain fatty acid levels, thereby providing anti-ALI effects.

In this investigation, there existed a pronounced correlation between the host’s amino acid and lipid metabolism and the composition of the intestinal microbiota. Correlation analyses integrating gut bacterial community profiles, fecal metabolomics, and pharmacological indices from lung and intestinal tissues revealed significant associations between pro-inflammatory mediators IL-1β, TNF-α, and IL-6 in the pulmonary environment of ALI mice and fecal metabolites such as tryptophan, L-kynurenine, formyl-kynurenine, arachidonic acid, leukotriene C4, and 12-HpETE. Furthermore, these mediators showed notable correlations with the abundance of Firmicutes, Lactobacillus, and Limosilactobacillus. Additionally, significant correlations were found between the level of oxidative stress in lung tissues and the presence of arachidonic acid, L-kynurenine, and formyl-kynurenine, as well as substantial associations with the abundance of Firmicutes, Lactobacillus, and Limosilactobacillus. In this study, the metabolic pathways of tryptophan were found to be crucial in modulating LPS-induced ALI in mice through the lung-gut axis, influenced by RRFJ. Tryptophan has the ability to modulate inflammation and immune responses within the organism. Depending on environmental conditions, tryptophan can follow different metabolic pathways, producing distinct end products. Tryptophan is enzymatically converted by IDO to form kynurenine. Additionally, during metabolic processes conducted by intestinal microbes, especially those from the Lactobacillus genus, tryptophan is converted into indole. Activated indole can bind to the AhR receptor in the intestinal environment, promoting epithelial cell regeneration, strengthening the intestinal barrier, and indirectly inhibiting the NF-κB signaling pathway, thereby reducing the production of pro-inflammatory cytokines, including IL-6. Arachidonic acid is a key biomarker in the pulmonary tissues of ALI mice, with its metabolites, such as leukotriene C4 and 12-HpETE, closely associated with the inflammatory cascade in ALI models. Intestinal bacteria ferment dietary fiber in the gastrointestinal tract, producing short-chain fatty acids (SCFAs) as a result. Higher levels of Firmicutes and Lactobacillus are positively correlated with increased SCFA production in the intestine, which helps to suppress the growth of pathogenic bacteria in the gut ecosystem. Moreover, SCFAs can reduce both inflammatory responses and oxidative stress levels in ALI-afflicted mice. Therefore, it is hypothesized that RRFJ might offer protection against LPS-induced ALI by modulating the lung-gut axis.

Through correlation analysis encompassing the gut bacterial community, fecal metabolomics, and pharmacological indices of lung and intestinal tissues, it was discerned that pro-inflammatory mediators IL-1β, TNF-α, and IL-6 within the pulmonary milieu of ALI-afflicted mice exhibited significant associations with fecal metabolites including tryptophan, L-kynurenine, formyl-kynurenine, arachidonic acid, leukotriene C4, and 12-HpETE, alongside marked correlations with the abundance of Firmicutes, Lactobacillus, and Limosilactobacillus. Furthermore, there existed significant correlations between the degree of oxidative stress observed within lung tissues and the presence of arachidonic acid, L-kynurenine, and formyl-kynurenine, as well as substantial associations with the abundance of Firmicutes, Lactobacillus, and Limosilactobacillus. Within the context of this investigation, the metabolic pathways of tryptophan played a pivotal role in modulating LPS-induced ALI in mice via the lung-gut axis, under the influence of RRFJ. Tryptophan possesses the capability to modulate inflammation and immune responses within the organism. Under varying environmental conditions, tryptophan may undergo differential metabolic pathways yielding distinct end products. Tryptophan undergoes enzymatic conversion catalyzed by IDO, resulting in the formation of kynurenine. Moreover, within the metabolic processes orchestrated by intestinal microbes, particularly those belonging to the Lactobacillus genus, tryptophan is transformed into indole. Indole, upon activation, can engage the AhR receptor located in the intestinal milieu, consequently fostering epithelial cell regeneration, fortifying the integrity of the intestinal barrier, and indirectly impeding the NF-κB signaling pathway, thereby mitigating the generation of pro-inflammatory cytokines, including IL-6. arachidonic acid emerges as a pivotal biomarker within the pulmonary tissues of ALI-afflicted mice, with its subsequent metabolites, including leukotriene C4 and 12-HpETE, intricately entwined with the inflammatory cascade in ALI murine models. Intestinal bacteria ferment dietary fiber within the gastrointestinal tract, yielding short-chain fatty acids (SCFAs) in the process. Elevated levels of Firmicutes and Lactobacillus abundance correlate positively with heightened SCFA production in the intestinal milieu, consequently impeding the proliferation of pathogenic bacterial strains within the gut ecosystem. Furthermore, SCFAs exhibit the capacity to mitigate both inflammatory responses and oxidative stress levels in ALI-afflicted murine models. Consequently, it is hypothesized that RRFJ may confer protection against LPS-induced ALI via modulation of the lung-gut axis.

**Figure 7:**
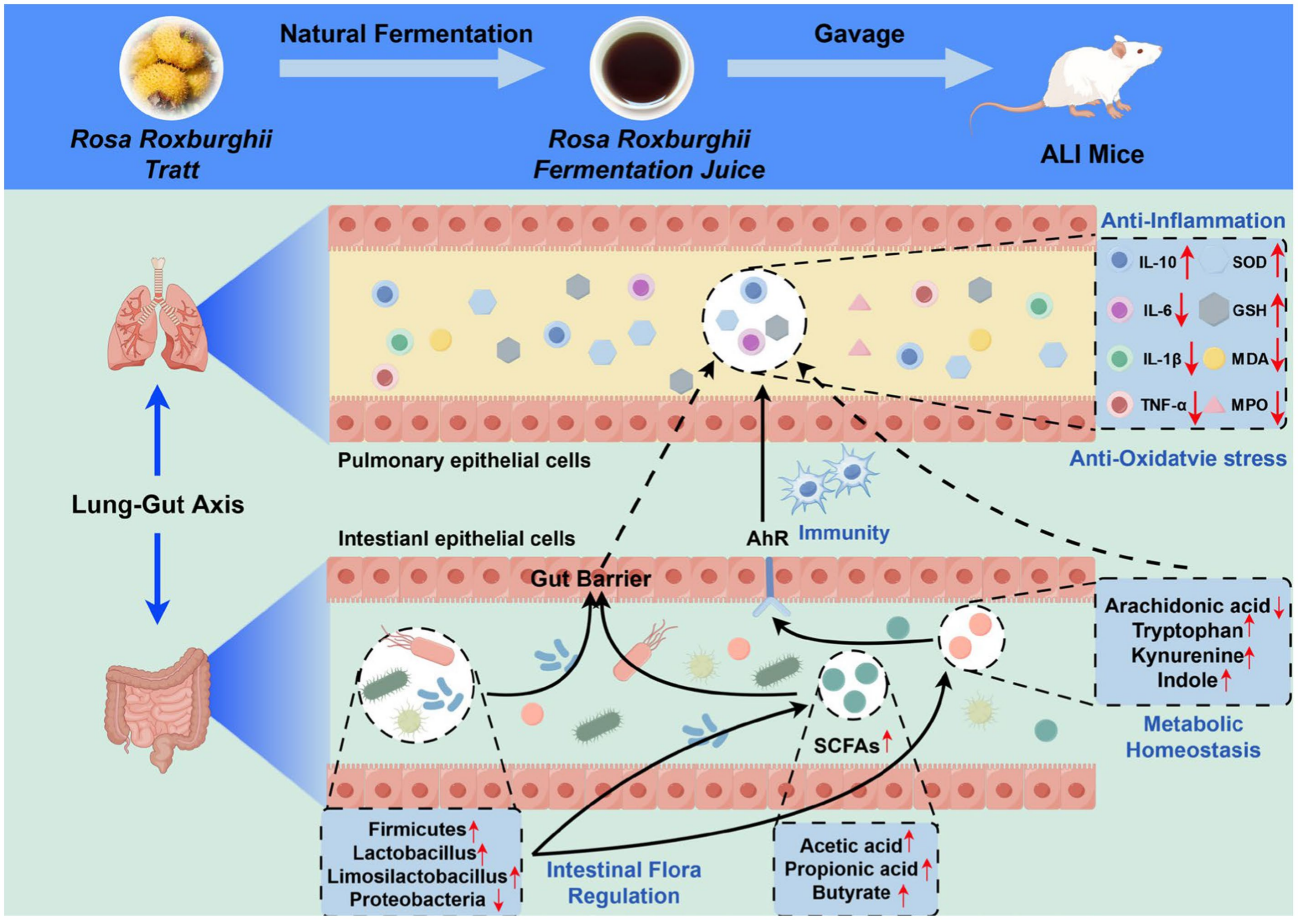
Schematic diagram representing the improvement mechanism of RRFJ in LPS induced ALI mice (by Figdraw).

In summary, this study represents the first comprehensive integration of intestinal microbiota and fecal metabolomics within the conceptual framework of the lung-gut axis to systematically elucidate the mechanism by which RRFJ alleviates LPS-induced ALI in mice (Figure 7). The results indicate that RRFJ corrects dysbiosis between probiotics and pathogenic bacteria by modulating amino acid and lipid metabolism in mice, thereby alleviating ALI. Additionally, correlation analyses demonstrate a strong association between pharmacological indices of lung tissue and both the composition of intestinal microbiota and specific intestinal metabolites. We propose that RRFJ has potential as an adjunctive therapy for ALI.

## Data Availability

The original contributions presented in the study are included in the article/supplementary material, further inquiries can be directed to the corresponding author.

## Footnotes

^1^https://www.genome.jp/kegg/

^2^http://www.hmdb.ca/
